# Exposure-Specific and Age-Specific Attack Rates for Ebola Virus Disease in Ebola-Affected Households, Sierra Leone

**DOI:** 10.3201/eid2208.160163

**Published:** 2016-08

**Authors:** Hilary Bower, Sembia Johnson, Mohamed S. Bangura, Alie Joshua Kamara, Osman Kamara, Saidu H. Mansaray, Daniel Sesay, Cecilia Turay, Francesco Checchi, Judith R. Glynn

**Affiliations:** London School of Hygiene & Tropical Medicine, London, UK (H. Bower, J.R. Glynn);; Save the Children, Freetown, Sierra Leone (S. Johnson, M.S. Bangura, A.J. Kamara, O. Kamara, S.H. Mansaray, D. Sesay, C. Turay);; Save the Children, London (F. Checchi)

**Keywords:** Ebola, Ebola virus, Ebola virus disease, viruses, attack rates, risk factors, exposures, transmission, survivors, households, cohort study, Sierra Leone, infectious disease transmission

## Abstract

Using histories of household members of Ebola virus disease (EVD) survivors in Sierra Leone, we calculated risk of EVD by age and exposure level, adjusting for confounding and clustering, and estimated relative risks. Of 937 household members in 94 households, 448 (48%) had had EVD. Highly correlated with exposure, EVD risk ranged from 83% for touching a corpse to 8% for minimal contact and varied by age group: 43% for children <2 years of age; 30% for those 5–14 years of age; and >60% for adults >30 years of age. Compared with risk for persons 20–29 years of age, exposure-adjusted relative risks were lower for those 5–9 (0.70), 10–14 (0.64), and 15–19 (0.71) years of age but not for children <2 (0.92) or 2–4 (0.97) years of age. Lower risk for 5–19-year-olds, after adjustment for exposure, suggests decreased susceptibility in this group.

## Abstract 2

Risk for disease correlated with level of exposure and was lowest for children 5–19 years of age.

In Ebola epidemics in West Africa and elsewhere, children appear to have been relatively spared (*1**–**5*). Published notification data for the West Africa outbreak that began in 2013 show a linear increase in incidence of Ebola virus disease (EVD) with age in persons up to ≈35 years of age, followed by a plateau in incidence for older age groups (*6*). Among children, the World Health Organization has reported a slightly increasing incidence with increasing age in Liberia and Sierra Leone but no clear pattern in Guinea (*4*). In contrast, published case-fatality rates for EVD are lowest for persons 10–15 years of age and highest for young children and older adults (*4**,**7*).

These age patterns could result from bias in recognizing, diagnosing, or reporting cases; differences in exposure; or differences in susceptibility to disease. Official data from the West Africa outbreak are known to be inaccurate (*8**,**9*). In previous, smaller outbreaks, case ascertainment could have been more complete because of the smaller scale, but EVD cases might have been missed, especially mild cases; deaths may also have been missed because the elderly and very young are more likely to sicken and die from other causes. Children with fever are less likely than adults to visit health facilities for care, and children may be underreported as contacts (*10*).

Exposure patterns are likely to differ by age and sex. Women may be more at risk from caring for the sick and men from carrying sick persons to the hospital. Children may be deliberately kept away from sick persons and funeral rites, and lower incidence among children has been attributed to these factors (*1**,**11*). However, preventing exposure of young children in Ebola-affected households is difficult. Children need to be held, fed, and cared for and often share beds with adults or other children; they may also be exposed through breastfeeding (*12*).

The high case-fatality rate observed in children <5 years of age and especially in those <1 year of age (*4*) suggests that young children are particularly susceptible to Ebola; consequently, low incidence in young children may reflect low exposure or low ascertainment. In a study of 27 Ebola-affected households after the Kikwit outbreak in 1995, children <18 years of age had lower risks of disease than adults, after adjustment for reported exposures (*13*).

Assessing whether risk by age depends on exposure or susceptibility requires a comparison of exposures in persons with and without EVD. A recent systematic review of risk factors for transmission of Ebola virus found few studies reporting data on risks (*14*) and no previous study large enough to stratify in detail by age (*3**,**13**,**15**–**19*). We interviewed a large cohort of EVD survivors and their household members to determine exposure levels of all members, living and dead, and to calculate attack rates and relative risks by age, sex, and type of exposure. The Sierra Leone Ethics and Scientific Review Committee and the Ethics Committee of the London School of Hygiene and Tropical Medicine approved the study.

## Methods

All survivors who were discharged from Kerry Town Ebola Treatment Centre (ETC), Sierra Leone, during November 2014–March 2015 and who lived in the Western Area were eligible for the study. During July–August 2015, members of the study team, which had assisted in survivor reintegration into the community, contacted survivors or their parents or guardians and asked them to bring all household members who were present at the time Ebola was affecting their household to an interview to be conducted at 1 of various locations. To make contact, the field team went to addresses of survivors when addresses were available and complete enough to locate or used telephone numbers when available. Team members were university graduates, nurses, and paramedics and included Ebola survivors; they received extensive training in interview techniques and were supervised by the first 2 authors (H.B. and S.J.), 1 or both of whom attended all interviews.

After obtaining individual written informed consent from each participant or parents or guardians of participants <18 years of age, the interviewers compiled a list of all members in each household and included information on age, sex, and household members who had had EVD and those who had died of EVD. Households were defined as persons eating from the same pot at the time EVD was in the household, regardless of how much time had been spent in the household, and included persons who joined the household to assist someone who was ill.

We asked household members to describe in their own words what had occurred in the household. For each person reported as having had EVD, we asked what symptoms occurred at home and which persons had helped that person during his or her EVD illness, shared a bed or had contact with the person, or had contact with the body if the person died. Adults spoke for young children and corroborated information from older children. Using probing questions and predefined exposure levels, we assigned a maximum exposure for persons who had been present in the household. The levels, which we developed on the basis of the literature and discussion with ETC staff, included touching the corpse of someone who died from EVD; direct contact with body fluids of a wet patient (i.e., with diarrhea, vomiting, or bleeding); direct contact with a wet patient; direct contact with a dry patient (i.e., without diarrhea, vomiting, or bleeding); indirect contact with a wet patient (e.g., washing clothes); indirect contact with a dry patient; minimal contact (e.g., shared meals); or no known contact (Table 1). We also asked about exposures outside the home and classified these exposures by using the same scale. For those reported as not having had EVD, we asked about any symptoms at the same time that others in the household had EVD. Study team members, all of whom are multilingual, conducted interviews in the participants’ language and recorded key outcomes in English.

**Table 1:** Classification of level of exposure to EVD patients in study of EVD risk for household members, Sierra Leone, 2014–2015*

Level	**Definition**
1	Contact with the body of EVD patient after death/prepared the body for burial
2	Direct contact with body fluids (e.g., blood, diarrhea, vomit, urine, or a baby breastfed by an EVD-positive woman)
3	Direct close contact with wet case; i.e., with diarrhea/vomiting/bleeding (e.g., person helped dress, embraced, carried, helped care for, or shared bed of an EVD patient with wet symptoms; or mother breastfed an EVD-positive child)
4	Direct close contact with dry case (i.e., without wet symptoms at the time) (e.g., person helped dress, embraced, carried, helped care for, or shared bed with an EVD patient without wet symptoms)
5	Indirect close contact with wet case (e.g., washed clothes or bed linen of an EVD patient with wet symptoms, or slept in the same room but not the same bed)
6	Indirect close contact with dry case (e.g., person washed clothes or bed linen of EVD patient without wet symptoms); formal/informal health workers without known contact with an EVD patient; ETC workers in PPE; Ebola Intervention workers (outside household only); person attended funeral without contact with the body (outside household only)
7	Minimal contact (e.g., person shared meals or utensils or sat In the same room; children placed in observation centers [outside household only])
8	No actual contact (e.g., person kept distance once EVD patient was symptomatic)

*ETC, Ebola Treatment Centre; EVD, Ebola virus disease; PPE, personal protective equipment.

### Definitions

Laboratory-confirmed EVD survivors who were reported from Kerry Town ETC, survivors from other ETCs, and all persons reported by the family as having died of EVD were counted as EVD case-patients. Deaths for which the family was unsure of the cause and symptomatic persons who were not tested or did not receive a diagnosis of EVD were classified as probable EVD case-patients if they fit the Sierra Leone case definition for probable cases (*20*).

For each household, the first person who became ill was identified as the likely primary case-patient. Some households reported 2 people who became ill at the same time, and they are counted as co-primary case-patients. No household described >1 period when Ebola occurred in the household. To avoid overburdening participants, we did not collect time sequences or dates and defined all nonprimary case-patients in a household as subsequent case-patients.

### Analysis

Our initial descriptive analysis of outcomes by age and sex included all household members. We subsequently analyzed primary case-patients separately because their exposure occurred outside the household, and we compared their characteristics with those of all other household members.

In the analysis of risks for disease by age, sex, and exposure level, we excluded primary case-patients and household members who were alive but not present for the interview and unable to consent to individual data collection. We explored the following variables for their effects on disease risk and as confounders of the associations of other variables and disease risk: having a spouse who contracted EVD first; occupation; being household head versus household member; and household-level variables (i.e., household size; crowding [number of persons/number of rooms]; and access to water, soap, and latrine). Our analysis used logistic regression and adjusted for household clustering by using random effects. Because risks were large, we used marginal standardization to estimate risk ratios (RRs) and the delta method to estimate 95% CIs (*21**,**22*). All analyses used Stata 14 (http://www.stata.com). We also performed a sensitivity analysis that excluded case-patients and deaths classified as probable EVD cases. 

## Results

### Study Population

**Figure 1:**
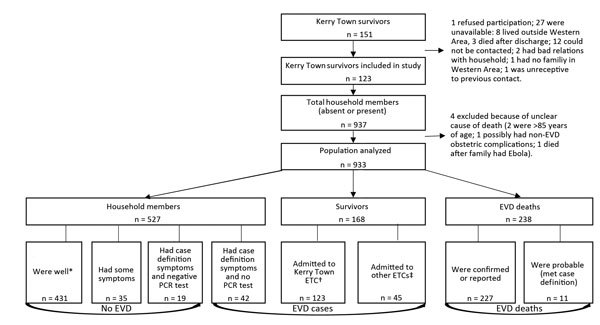
Flow diagram showing the population composition for study of Ebola-affected households related to survivors from the Kerry Town Ebola Treatment Centre (ETC), Sierra Leone, 2014–2015. EVD, Ebola virus disease. *Includes 23 not present for interview. †Includes 1 who died after discharge. ‡Includes 5 not present for interview.

Of 151 EVD survivors discharged from Kerry Town ETC, we included 123 survivors from 94 households in the study. The other 28 survivors had a similar age distribution to those included (39% of survivors not included vs. 36% of those included were <15 years of age) and a slightly higher proportion of males (54% of those not included vs. 38% of included survivors). We collected detailed information for 937 persons, including exposure histories for 909 (Figure 1).

Overall, 448 persons were reported as having had EVD or probable EVD, of whom 238 (53%) died; 227 deaths were reported as caused by EVD, and the 11 other deaths fit the EVD case definition. Among survivors, 123 were EVD patients at the Kerry Town ETC, and 45 were at other ETCs. An additional 42 household members had probable EVD; the remaining 485 household members had no evidence of EVD.

**Figure 2:**
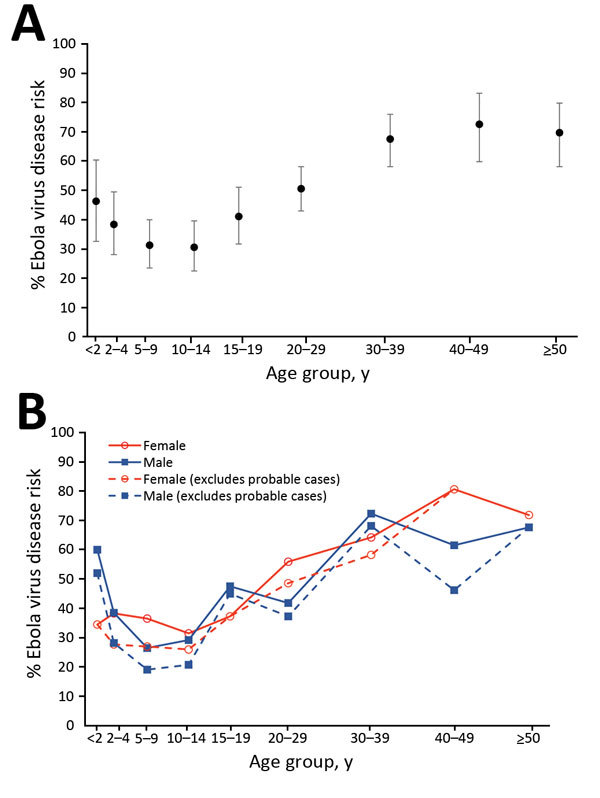
Risk for Ebola virus disease in Ebola-affected households of Kerry Town Ebola Treatment Centre survivors, by age and sex, Sierra Leone, 2014–2015. A) Risk by age group; bars indicate 95% CIs. B) Risk by sex and age group with and without probable cases.

Risk for EVD was lowest for children 5–14 years of age but higher for children <2 years of age and for adults (Table 2). Risk increased with age for adults up to ≈35 years of age and then plateaued for older adults (Figure 2, panel A). Because most probable case-patients were children, the lower risk for children was more extreme when probable case-patients were excluded (Table 2). EVD risk was similar for male and female study participants, even when results were stratified by age (Figure 2, panel B).

**Table 2:** Distribution of outcomes by age and sex among Kerry Town Ebola Treatment Centre survivors and their household members, Sierra Leone, 2014–2015*

Characteristic	Total	No. (%)							Overall % EVD#
		Persons with no symptoms	Persons with some symptoms†	SympCD and neg test‡	SympCD and no test§	EVD survivors	Probable EVD deaths¶	EVD deaths	
Total	933	431 (46.2)	35 (3.8)	19 (2.0)	42 (4.5)	168 (18.0)	11 (1.2)	227 (24.3)	48
Sex									
M	399	184 (46.1)	19 (4.8)	11 (2.8)	20 (5.0)	62 (15.5)	5 (1.3)	98 (24.6)	46
F	534	247 (46.3)	16 (3.0)	8 (1.5)	22 (4.1)	106 (19.9)	6 (1.1)	129 (24.2)	49
Age, y**									
<2	54	27 (50.0)	2 (3.7)	0	1 (1.9)	4 (7.4)	1 (1.9)	19 (35.2)	46
2–4	86	49 (57.0)	2 (2.3)	2 (2.3)	8 (9.3)	9 (10.5)	1 (1.2)	15 (17.4)	38
5–9	131	82 (62.6)	4 (3.1)	4 (3.1)	11 (8.4)	15 (11.5)	0 (0.0)	15 (11.5)	31
10–14	121	78 (64.5)	3 (2.5)	3 (2.5)	8 (6.6)	18 (14.9)	0	11 (9.1)	31
15–19	107	57 (53.3)	4 (3.7)	2 (1.9)	1 (0.9)	28 (26.2)	0	15 (14.0)	41
20–29	178	76 (42.7)	8 (4.5)	4 (2.2)	10 (5.6)	49 (27.5)	1 (0.6)	30 (16.9)	51
30–39	114	31 (27.2)	3 (2.6)	3 (2.6)	3 (2.6)	26 (22.8)	3 (2.6)	45 (39.5)	68
40–49	62	12 (19.4)	4 (6.5)	1 (1.6)	0	12 (19.4)	4 (6.5)	29 (46.8)	73
>50	76	18 (23.7)	5 (6.6)	0	0	7 (9.2)	0	46 (60.5)	70

*Excluded are 4 persons who died with uncertain cause (3 females, 1 male). EVD, Ebola virus disease. †Persons had some EVD symptoms but did not fulfill case definition. ‡Persons met EVD case definition on interview but reported a negative PCR test for Ebola. §Persons met EVD case definition on interview but were never tested.  ¶Description of symptoms leading to death were compatible with Ebola, but EVD was not diagnosed at the time.  #Overall % EVD includes EVD cases and deaths, SympCD and no test, and Probable EVD deaths as case-patients.  **Age missing for 4 persons (2 reported EVD deaths, 1 probable EVD death, 1 with no symptoms).

### Primary Case-Patients

Primary case-patients were identified for 91 households and co-primary case-patients in 3 households. Compared with all other household members, primary case-patients were older, usually >30 years of age; slightly more likely to be male; and more likely to be household heads, healthcare or EVD front-line workers, or religious or community leaders (Table 3). Children or students were least likely to be primary case-patients. In 5 households, primary case-patients joined the household when they were already ill.

**Table 3:** Risk factors associated with being the first EVD case in a household, compared with all other household members in households of Ebola Treatment Centre survivors, Kerry Town, Sierra Leone, 2014–2015*

Risk factor	Total population	No. primary cases	Risk,%	Adjusted RR* (95% CI)	p value†
Sex					
M	400	47	11.8	1.3 (0.93–1.9)	
F	537	50	9.3	1	0.1
Age, y‡					
<2	54	3	5.6	0.57 (0.17–1.9)	
2–4	86	2	2.3	0.24 (0.06–1.0)	
5–9	131	2	1.5	0.15 (0.04–0.65)	
10–14	121	3	2.5	0.26 (0.08–0.86)	
15–19	107	7	6.5	0.69 (0.30–1.6)	
20–29	179	17	9.5	1	
30–39	114	26	22.8	2.4 (1.4–4.2)	
40–49	63	15	23.8	2.5 (1.3–4.7)	
>50	78	20	25.6	2.6 (1.5–4.7)	<0.001
Occupation§					
Heathcare worker, formal and informal	21	10	47.6	3.9 (2.0–7.5)	
Ebola front-line worker	11	3	27.3	2.5 (0.86–7.1)	
Driver	23	6	26.1	1.7 (0.67–4.1)	
Religious leader/chief/teacher	12	5	41.7	2.7 (1.1–6.9)	
Farmer/fisherman/unskilled	54	12	22.2	1.7 (0.83–3.3)	
Office/business	47	8	17.0	1.3 (0.62–2.8)	
Child/student	511	15	2.9	0.41 (0.12–1.4)	
Trader/tailor/service	205	25	12.2	1	0.01
Position in household					
Household head	87	32	36.8	2.3 (1.5–3.8)	
Household member	850	65	7.6	1	<0.001

*EVD, Ebola virus disease; RR, risk ratio. Adjusted for age, sex, and household clustering. †p values calculated from likelihood ratio test in logistic regression model. ‡Age missing for 4 persons. §No occupation recorded for 29 persons, including 28 with no individual-level data.

Likely sources of infection were identified for 68 (70%) of 97 primary case-patients. When >1 source of infection was possible, we selected the highest exposure level (Table 1). Thirty primary case-patients visited a household with an EVD patient; 16 of those 30 went to help the ill patient. Eight prepared bodies for burial or touched the corpse; 6 attended funerals; 4 carried a person with EVD symptoms; 8 attended healthcare facilities; and 12 worked as healthcare or front-line workers, 5 of whom were known to have treated an EVD patient.

### Subsequent Case-Patients

The overall risk for acquiring EVD was 43% and was similar for male and female participants (Table 4); the risk by age was J-shaped, as for the full study population. Among household members, 60% reported direct contact with a wet patient or their fluids or with a person who died of EVD (Table 4). Only 10 (1.2%) household members had a substantially higher level of exposure outside the household than inside.

**Table 4:** Risk factors associated with development of EVD in subsequent case-patients in Ebola-affected households, Kerry Town, Sierra Leone, 2014–2015*

Risk factor	No. patients/no. total (%),† N = 809	Adjusted RR‡ (95% CI)	Adjusted RR§ (95% CI)	Multivariable RR¶ (95%CI)	p value#
Sex					
M	136/337 (40.4)	1.1 (0.87–1.4)	1.06 (0.85–1.3)	1.03 (0.87–1.2)	
F	211/472 (44.7)	1	1	1	0.7
Age, y					
<2	22/51 (43.1)	0.79 (0.49–1.3)	0.80 (0.49–1.3)	0.92 (0.64–1.3)	
2–4	31/81 (38.3)	0.70 (0.45–1.1)	0.70 (0.46–1.1)	0.97 (0.72–1.3)	
5–9	38/127 (29.9)	0.44 (0.28–0.68)	0.44 (0.28–0.69)	0.70 (0.50–0.97)	
10–14	34/114 (29.8)	0.41 (0.25–0.67)	0.41 (0.25–0.67)	0.64 (0.45–0.93)	
15–19	35/95 (36.8)	0.53 (0.33–0.84)	0.53 (0.33–0.84)	0.71 (0.49–1.02)	
20–29	72/155 (46.5)	1	1	1	
30–39	51/85 (60.0)	1.2 (0.82–1.6)	1.2 (0.82–1.6)	1.1 (0.83–1.4)	
40–49	30/46 (65.2)	1.2 (0.82–1.8)	1.2 (0.83–1.8)	1.1 (0.80–1.6)	
>50	33/53 (62.3)	1.3 (0.87–1.8)	1.3 (0.88–1.8)	1.3 (0.97–1.8)	0.004
Maximum exposure					
Handled corpse	60/72 (83.3)	18.1 (7.4–44.1)	13.5 (5.4–33.5)	11.1 (4.5–27.4)	
Handled fluids	73/120 (60.8)	13.1 (5.4–31.9)	9.7 (3.9–24.1)	8.5 (3.5–20.6)	
Direct wet contact	146/297 (49.2)	10.4 (4.3–25.1)	8.3 (3.4–20.1)	7.1 (3.0–17.1)	
Direct dry contact	47/125 (37.6)	7.1 (2.9–17.7)	5.6 (2.3–13.9)	5.3 (2.2–12.9)	
Indirect wet contact	5/19 (26.3)	5.7 (1.6–20.1)	4.9 (1.4–16.8)	4.7 (1.5–14.6)	
Indirect dry contact	8/74 (10.8)	1.4 (0.43–4.6)	1.3 (0.40–4.2)	1.3 (0.41– 4.0)	
Minimal/no contact**	8/102 (7.8)	1	1	1	<0.001
Position in household					
Household head	24/52 (46.2)	1.2 (0.79–1.79)	0.62 (0.35–1.1)	0.62 (0.39–1.0)	
Household member	323/757 (42.7)	1	1	1	0.03
Household size					
>16	120/209 (57.4)	5.2 (1.6–16.9)	5.0 (1.5–16.8)	2.9 (1.1–7.8)	
11–15	98/290 (33.8)	2.4 (0.70–7.9)	2.4 (0.70–8.3)	1.7 (0.63–4.6)	
6–10	121/270 (44.8)	3.7 (1.2–12.0)	3.9 (1.2–12.8)	2.7 (1.04–7.0)	
1–5	8/40 (20.0)	1	1	1	0.01
Occupation					
HCW (formal and informal)	8/11 (72.7)	1.6 (1.1–2.5)	1.8 (0.91–3.6)		
Ebola front-line worker	1/8 (12.5)	0.16 (0.02–1.5)	0.14 (0.02–1.3)		
Driver	11/17 (64.7)	1.2 (0.67–2.0)	1.1 (0.55–2.3)		
Religious leader/chief/teacher	5/7 (71.4)	1.6 (0.95–2.8)	1.8 (0.70–4.4)		
Farmer/fisherman/unskilled	18/41 (43.9)	0.81 (0.49–1.4)	0.85 (0.48–1.5)		
Office/business	26/39 (66.7)	1.3 (0.89–1.8)	1.4 (0.87–2.2)		
Child/student	173/484 (35.7)	0.52 (0.40–0.69)	1.0 (0.63–1.6)		
Trader/tailor/service	96/177 (54.2)	1	1		
Water available					
Sometimes	45/131 (34.4)	0.58 (0.2–-1.3)	0.59 (0.28–1.3)		
Most days	118/265 (44.5)	1.0 (0.59–1.59)	0.96 (0.60–1.5)		
Every day	182/408 (44.6)	1	1		
Soap available					
Sometimes	64/194 (33.0)	0.78 (0.42–1.4)	0.84 (0.47–1.5)		
Most days	113/200 (56.5)	1.5 (0.90–5.5)	1.4 (0.84–2.3)		
Every day	168/410 (41.0)	1	1		
Latrine					
Household’s own	107/286 (37.4)	0.7(0.43–1.2)	0.72 (0.43–1.2)		
Shared/none	238/518 (45.9)	1	1		
Crowding					
High	126/238 (52.9)	2.1 (0.89–4.7)	2.4 (1.0–5.4)		
Medium	189/483 (39.1)	1.4 (0.63–3.2)	1.6 (0.71–3.6)		
Low	30/83 (36.1)	1	1		
Spouse with Ebola first					
Yes	45/77 (58.4)	1.6 (1.2–2.1)	1.0 (0.68–1.5)		
No	302/732 (41.3)	1	1		

*Subsequent case-patients were any household members who contracted Ebola virus disease after the first (primary) case-patient. Data were excluded for 4 deaths from uncertain cause, 27 persons with no individual-level data, and 97 primary cases. Data were missing for 2 persons with no recorded age, 6 persons with no recorded occupation, and 5 persons (in 1 household) with no recorded information about water, soap, latrine, and crowding. EVD, Ebola virus disease; HCW, healthcare worker; RR, risk ratio. †Number of subsequent case-patients/total number of household members in study, excluding primary case-patients.  ‡Adjusted for household clustering. §Adjusted for age, sex, and household clustering. ¶Adjusted for clustering and all other factors included in the model. #p values for multivariable model calculated from likelihood ratio test in logistic regression model. **Only 7 persons reported no contact, so these 2 categories are combined.

**Figure 3:**
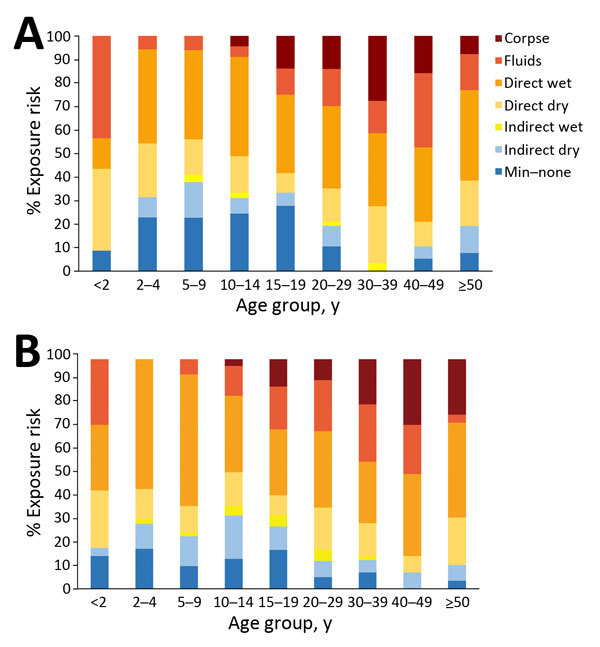
Levels of exposure to Ebola virus disease among households of Kerry Town survivors, excluding primary case-patients, by age and sex, Sierra Leone, 2014–2015. A) Male participants; B) female participants. Levels of exposure correspond to those shown in Table 1. Min–none, minimum or no exposure.

Attack rates increased steeply and linearly with the predefined exposure levels. Exposure levels were high at all ages and for males and females (Figure 3), but exposure to EVD corpses increased with age, and direct exposure to fluids was higher for children <2 years of age, largely because of breastfeeding, and for older adults. After adjustment for age and sex, attack rates varied by occupation and were higher in larger and more crowded households. We found no clear associations with household-level measures of sanitation nor with having a spouse who developed EVD first (Table 4).

A multivariable analysis (Table 4) showed that developing EVD as a subsequent case-patient was strongly associated with age (p = 0.004), level of exposure (p<0.001), not being a household head (p = 0.03), and household size (p = 0.01). Sex was kept in the model a priori but was not associated with EVD risk. Occupation was not associated with EVD risk after adjustment for exposure level (p = 0.2). In the full model, the association with age was still J-shaped. The lowest risk was for persons 5–19 years of age, and risks were higher for older than younger adults. Additional adjustment for other available variables had little effect on associations. In the sensitivity analysis that excluded probable EVD cases, associations with exposure levels were stronger, and the J-shaped association with age was more marked (Table 5).

**Table 5:** Sensitivity analysis showing risk factors associated with development of EVD as a subsequent case-patient in Ebola-affected households, Kerry Town, Sierra Leone, 2014–2015*

Risk factor	Total, excluding probable cases, N = 764		
	No. patients/no. total (%)†	Adjusted RR‡ (95% CI)	p value§
Sex			
M	114/315 (36.2)	1.0 (0.83–1.2)	
F	188/449 (41.9)	1	1.0
Age, y			
<2	21/50 (42.0)	0.99 (0.66–1.5)	
2–4	22/72 (30.6)	0.98 (0.68–1.4)	
5–9	27/116 (23.3)	0.69 (0.47–1.0)	
10–14	26/106 (24.5)	0.60 (0.39–0.93)	
15–19	34/94 (36.2)	0.77 (0.52–1.1)	
20–29	63/146 (43.2)	1	
30–39	47/81 (58.0)	1.2 (0.86–1.6)	
40–49	28/44 (63.6)	1.2 (0.82–1.8)	
>50	33/53 (62.3)	1.5 (1.1–2.0)	0.002
Maximum exposure			
Handled corpse	60/72 (83.3)	40.6 (8.5–194.5)	
Handled fluids	65/112 (58.0)	30.5 (6.4–144.8)	
Direct wet contact	125/276 (45.3)	24.1 (5.2–113.2)	
Direct dry contact	41/119 (34.5)	16.7 (3.6–78.1)	
Indirect wet contact	5/19 (26.3)	17.2 (3.1–94.7)	
Indirect dry contact	4/70 (5.7)	2.3 (0.37–14.3)	
Minimal/no contact	2/96 (2.1)	1	<0.001
Position in household			
Household head	22/50 (44.0)	0.58 (0.35–0.98)	
Household member	280/714 (39.2)	1	0.02
Household size			
>16	108/197 (54.8)	2.6 (0.98–6.7)	
11–15	90/282 (31.9)	1.5 (0.57–3.9)	
6–10	96/245 (39.2)	2.3 (0.89–5.7)	
1–5	8/40 (20.0)	1	0.04

*Subsequent case-patients were any household members who contracted EVD after the first (primary) case-patient. EVD, Ebola virus disease; RR, risk ratio. †Excluded data: 4 deaths from uncertain cause; 27 persons with no individual-level data, 97 primary case-patients; 45 case-patients were classified as having EVD on the basis of their histories but had no diagnosis of EVD at the time. Missing data: 2 persons with age unknown.  ‡Adjusted for clustering and all variables in the model. §p values calculated from likelihood ratio test in logistic regression model.

## Discussion

In Ebola-affected households in our study, the age pattern for EVD incidence in children differed from that reported for the overall epidemic by the World Health Organization (*4*) and was closer to the age pattern of reported case-fatality rates; children <5 years of age had higher risks than older children (*4**,**7*). Among adults, the pattern was similar to previous findings (*6*), with a plateau occurring >35 years of age. This pattern was similar whether probable case-patients were included or not. Children were less likely than adults to be primary case-patients, and among child primary case-patients, no particular trend by age was observed (Table 3). The higher risk for EVD among children <5 years of age than among older children may suggest that very young children have been disproportionately missed in notification data.

Our study included only survivor households because it was conducted by building on survivor-support links; consequently, it missed households with only fatal cases and those in which no one sought care. Compared with all Ebola-affected households, households in our study were likely to be larger; to include more EVD patients, which increases the chance that >1 household member survived; and to include more children >5 years of age, who have a lower case-fatality rate than younger children. These characteristics would tend to increase attack rates and may explain the high attack rate overall and the association of attack rate with household size. These characteristics might also increase the proportion of cases among children, although children >5 years of age had a relatively low incidence of EVD.

After excluding primary case-patients, we examined the extent to which age patterns could be explained by exposure levels. After we adjusted age-specific incidence data by exposure, children 5–19 years of age still had a lower risk for EVD, although the lower risk was less marked, and the increased risk with age for adults no longer plateaued but continued upward. If we measured exposure accurately, these findings suggest that some of the variation in risk by age within households results from differences in susceptibility. In the interviews, we avoided lengthy questionnaires with each person to try to reduce questionnaire fatigue, respondents’ forgetting or denying types of exposure, and possibly overburdening already traumatized households. Instead, we encouraged families to tell their stories, ensuring that we learned which household members had contact with each EVD patient and what type of contact. Consequently, the conversation flowed naturally, with different household members contributing and providing details, helping to minimize recall bias. This approach also enabled us to acquire details for children and for persons who had died, although use of proxy respondents may have limited accuracy of exposure measurement. We conducted the interviews 4–9 months after the illness, but participants provided considerable detail in their responses. Inaccuracies in recall would lead to a failure to adjust completely for exposure level, whereas any tendency to recall greater exposures for household members with EVD would increase the association with exposure and result in the association between age and EVD being overadjusted for exposure level.

We predefined exposure levels so that we could record only the highest level and not probe for details for possible lower levels. This approach differed from that of other studies (*13**,**14**,**16*), which recorded several exposures and adjusted during analysis. Our hierarchy of exposure appears to be accurate; we found strong correlations between EVD risk and each increase in exposure level.

As others have reported (*13**,**14**,**16*), the highest risk for EVD exposure was from contact with dead bodies. Risk was also high from direct contact with fluids and with wet patients and was lower but still considerable (5-fold [17-fold in the sensitivity analysis], compared with minimal risk) from direct contact with dry patients and indirect contact with wet patients (Tables 4,5). We found no discernible increase in risk from indirect contact with dry patients compared with exposures classified as minimal risk (Table 1). Overall, after exclusion of primary and co-primary case-patients, we found a high household attack rate, higher than found in previous studies (*23*), perhaps reflecting the urban setting and the bias toward households with multiple cases.

Children had lower exposure than adults, but exposure levels in these households were high overall; >50% of each age group had at least direct exposure to a wet patient. In the sensitivity analysis, correlation between exposure levels and outcome was stronger, suggesting misclassification of some case-patients included as probable EVD cases; this analysis also showed a markedly lower EVD risk in children >5 years of age.

A lower susceptibility to EVD among children is possible. Lower attack rates or case-fatality rates in children have been found for other viral diseases, including varicella (*24*), smallpox (*25*), and West Nile virus disease (*26*). For EVD, different cytokine and chemokine responses related to survival have been noted for adults and children (*27*).

We found little difference in risk by sex, even when stratified by age. Household-level measures of sanitation had surprisingly little effect on the outcome (*28*). Having a spouse who contracted EVD first was not a risk factor after we adjusted for age; consequently, sexual transmission did not appear to be an important factor in the acute phase.

We established likely sources of infection for 70% of primary case-patients. Although some were linked to high-risk activities, more were related to visits to friends and relatives, including some visits to nurse sick relatives. Other households were infected by taking in sick relatives. These activities show remarkable altruism at a stage of the epidemic when Ebola was well known. More support to families to protect themselves in the home when they helped those not known to have EVD might have prevented these transmissions.

Much of what we know about risks for Ebola virus transmission comes from anecdotal reports or case series (*29*). Few studies have measured risk associated with particular exposures directly (*13**,**15**,**16**,**18**,**23*), and none have been large enough to examine risk by age in detail. This study collected information on >800 contacts, enabling estimates of exposure-specific and age-specific attack rates. After we adjusted for exposure, age patterns for Ebola attack rates were similar to those for case-fatality rates. Inherent differences in susceptibility, which warrant further investigation, likely underlie both distributions.
